# Catatonia in depressive disorder, more usual than it is supposed to be

**DOI:** 10.1192/j.eurpsy.2023.1784

**Published:** 2023-07-19

**Authors:** R. J. Carrillo Molina, F. Vilchez Español, I. Caparros del Moral

**Affiliations:** Complejo Hospitalario universitario de Jaén, Jaén, Spain

## Abstract

**Introduction:**

Catatonia is a psychomotor syndrome characterized by various motor, affective and behavioral symptoms. It can occur as a cause of various underlying organic and psychiatric disorders. In Psychiatric nosology is used to specify a subtype of the disorder underlying.Unlike what was assumed in the past, today it is accepted that catatonia is more frequent in affective disorders than in schizophrenia. But despite this, diagnosis and treatment are still late in affective cases on many occasions.

**Objectives:**

-A case of catatonia is presented to review the diagnostic difficulties that can sometimes entail.-Review treatment algorithm.

**Methods:**

We present the case of a 62-year-old woman, initially diagnosed of major depressive symptoms with psychotic symptoms, showing no response to different treatments, evolving to catatonia, which is diagnosed after screening for neurological and medical diseases.

**Results:**

The patient had an adequate evolution after the withdrawal of antipsychotics and the application of ECT (Electroconvulsive therapy).

**Conclusions:**

- It is important to carry out an adequate screening, because many times the symptoms are caused by medical or neurological diseases.

-Catatonia has a good prognosis with an early treatment, but it may increase the risk of mortality after 5 days from the onset of symptoms.

-It is important to avoid the use of antipsychotics or other dopamine blockers. The use of benzodiazepines and ECT is indicated.

**Disclosure of Interest:**

None Declared

